# Supplementary material for: Genetic Bioaugmentation‐Mediated Bioremediation of Terephthalate in Soil Microcosms Using an Engineered Environmental Plasmid
Source: Microb Biotechnol. 2025 Jan 13;18(1):e70071. doi: 10.1111/1751-7915.70071 (PMC11725763; doi:10.1111/1751-7915.70071)
Supplement: Supplementary file 1 — Figures S1–S7. [file MBT2-18-e70071-s001.pdf]

Genetic bioaugmentation-mediated bioremediation of terephthalate in soil microcosms using an engineered environmental plasmid

Alejandro Marquiegui Alvaro[1], Anastasia Kottara[2], Micaela Chacón [1], Lisa Cliffe[1], Michael Brockhurst[2*], Neil Dixon[1*]

[1] Manchester Institute of Biotechnology (MIB), and Department of Chemistry, [2] School of Biological Sciences, The University of Manchester, Manchester, M1 7DN, UK

*Corresponding authors

michael.brockhurst@manchester.ac.uk
neil.dixon@manchester.ac.uk

Supplementary Information

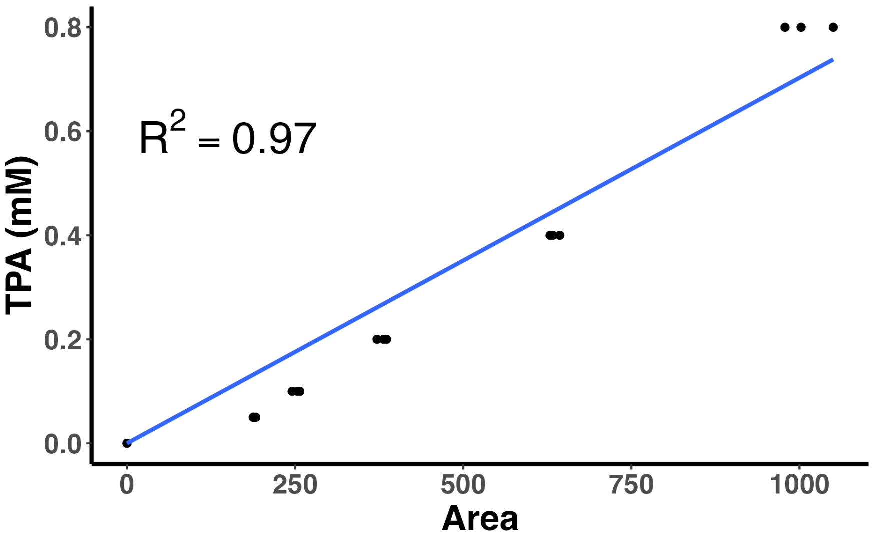

Supplementary Figure 1. TPA standard curve for HPLC

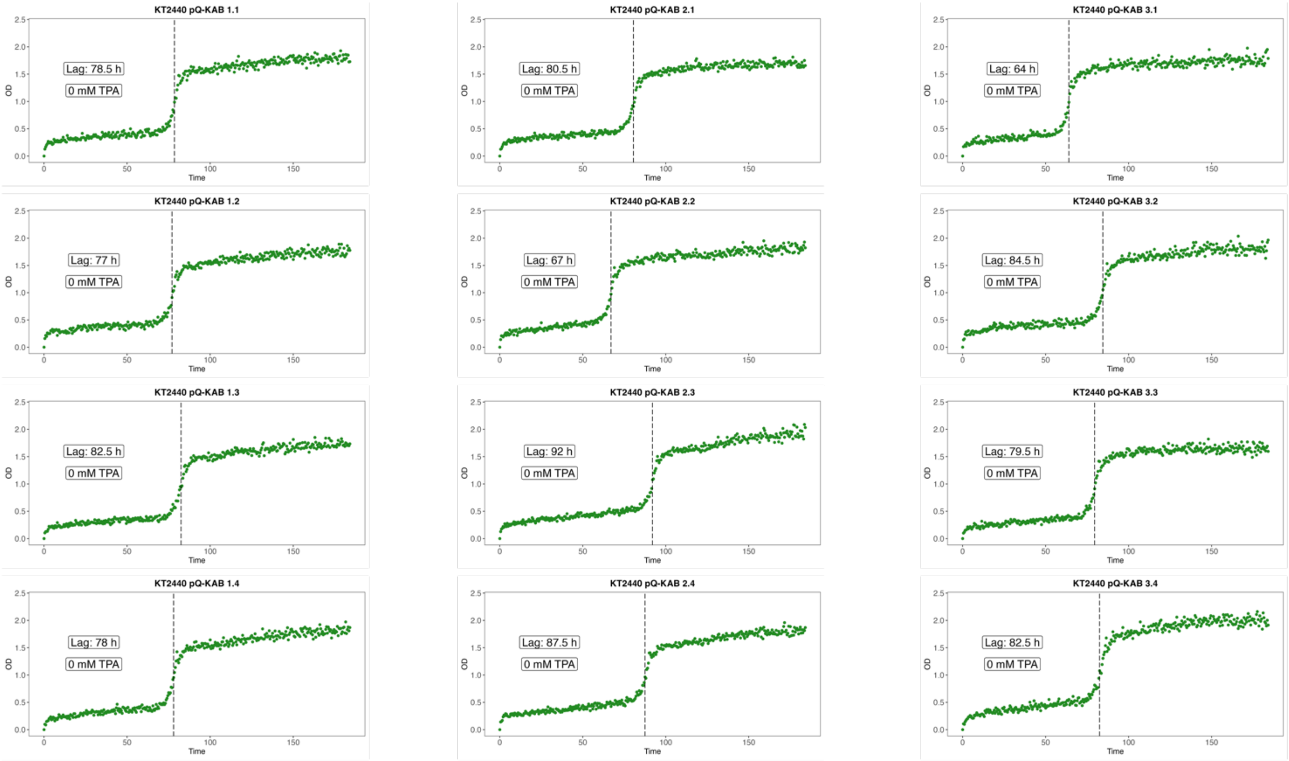

Supplementary Figure 2. *P. putida* pQBR57-KAB each individual replicate growth curves, with TPA as the sole carbon source, the lag-phase duration and final TPA concentration indicated.

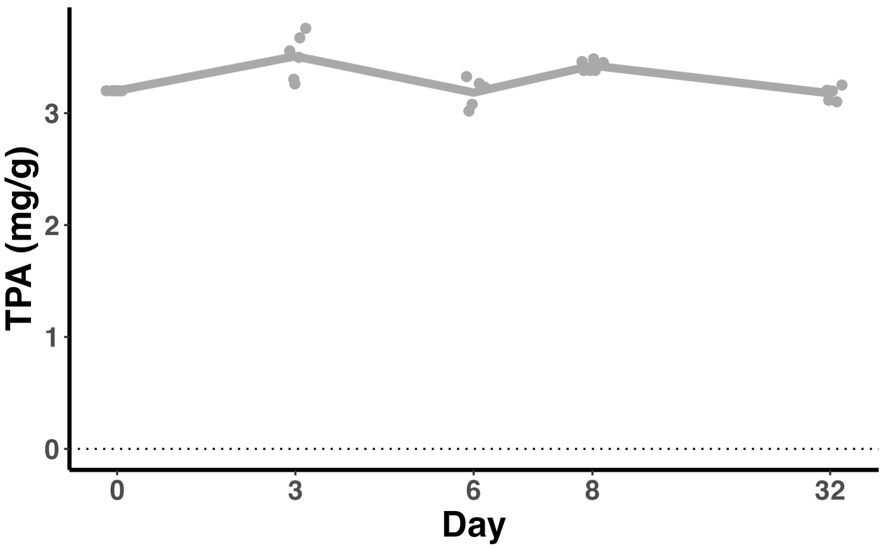

Supplementary Figure 3. Control of TPA degradation in soil, no bacteria were inoculated.

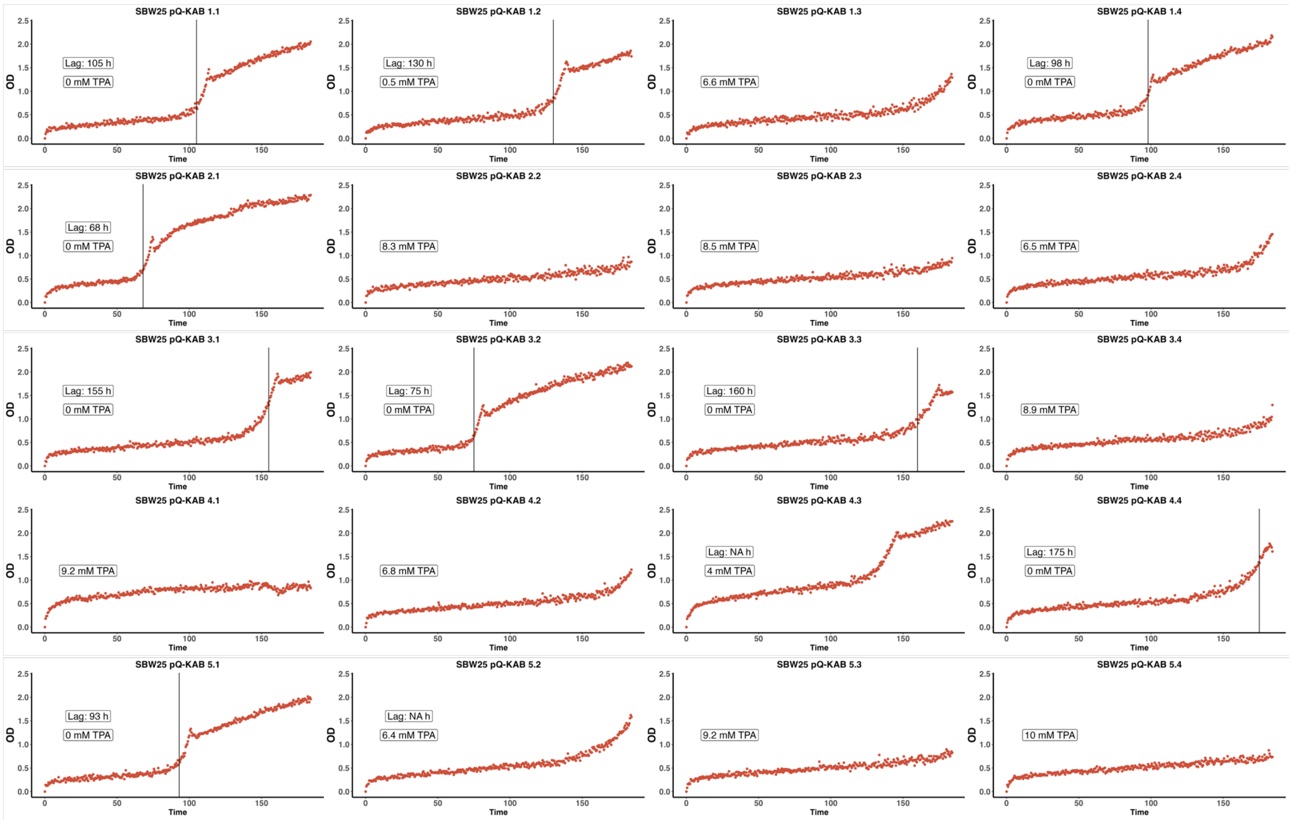

Supplementary Figure 4. Soil-isolated transconjugants of *P. fluorescens* pQBR57-KAB growing on M9 + 10mM TPA. The final concentration of TPA and the duration of the lag-phase are included in each plot.

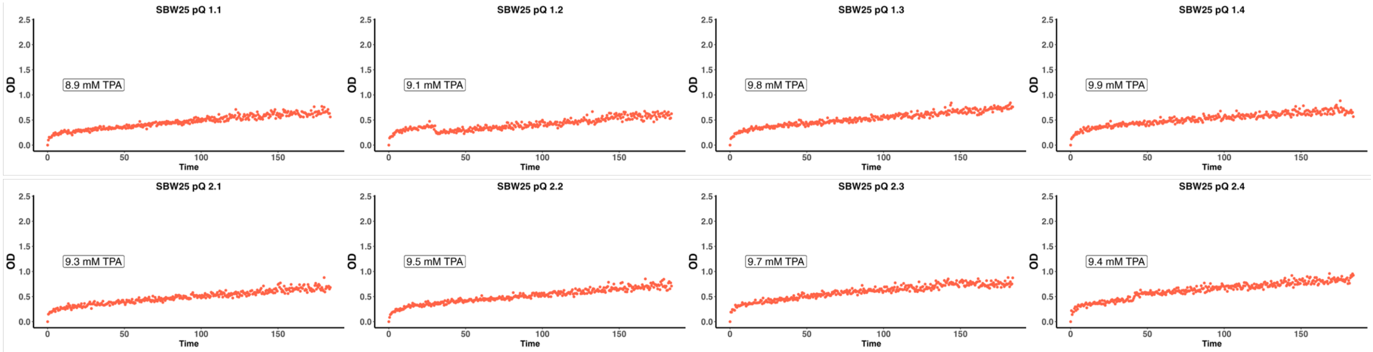

Supplementary Figure 5. Soil-isolated transconjugants of *P. fluorescens* pQBR57 growing on M9 + 10mM TPA. The final concentration of TPA is included in each plot.

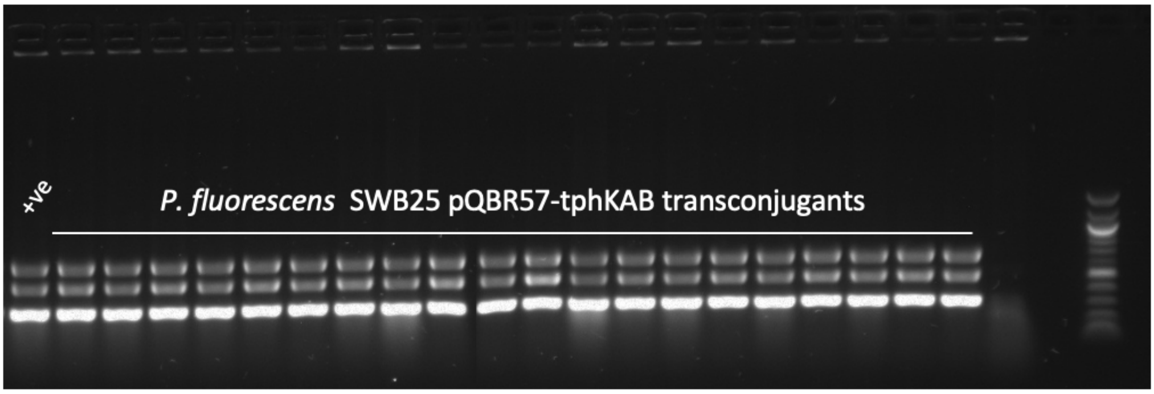

Supplementary Figure 6. PCR confirmation of soil-isolated transconjugants of *P. fluorescens* pQBR57-KAB. The positive control was *P. putida* pQBR57-KAB. The reaction is multiplexed and its targeting *merA*, *tphK* and *uvrD* in pQBR57-KAB.

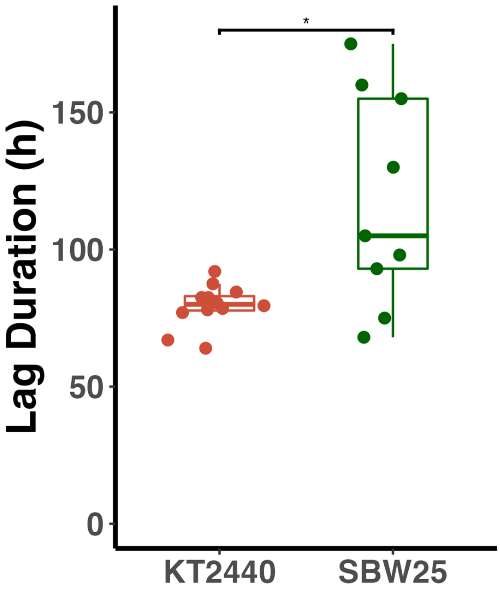

Supplementary Figure 7. Difference in lag duration of *P. putida* and *P. fluorescens* carrying pQBR57-KAB growing on M9 + 10 mM TPA.
